# Personal Experience

**Published:** 1916-11

**Authors:** L. P. Haskell

**Affiliations:** Chicago, Ill.


### PERSONAL EXPERIENCE.

DR. L. P. HASKELL, CHICAGO, ILL.

Occupying as I do a unique position in dental practice, I am more and more impressed with what has come to me in personal experience of importance to the profession.

For seventy years I have devoted my time to the construction of artificial dentures. For more than thirty years have worn partial upper and lower dentures, and full dentures. For twelve years have worn a denture on that problem of the mouth, "the flat lower jaw," with success, so have solved that problem.

I may safely say that this entire experience has never been duplicated in the mouth of another dentist, and unless so, no dentist could realize what I have, and learn what can be learned in relation to the articulation of teeth, and just what takes place in the use of them in eating; in the movement of the jaw (excuse me if I do not say mandible, a scientific fad), in the use of the lower lip (a very important thing), and the way the tongue acts, which is indescribable.

Patients always found it impossible to properly masticate tough meat, or any other tough substance, because such requires grinding, and it is impossible to do this except in favorable conditions, as for instance, an upper set fitted to a good ridge, with lower natural teeth and bridges, the movement of the jaw is simply a crushing one, up and down. The lower lip presses against the denture, holding it in place; this is greatly aided by the flanges beside the posterior teeth in the case of the flat jaw.

It is unfortunate that the dentist who has taken great pains to follow anatomical articulation, can not get inside the mouth, to see just what is going on there. The great mistake is, that the artificial teeth are articulated as natural ones are, allowing the anterior teeth to come in contact, overlooking the fact that the natural teeth are firm in the jaws, while the artificial teeth are on movable plates, often on flexible ridges, flat jaws, and jaws in changing condition. The anterior teeth should never come in contact, for sooner or later there is trouble.

One of the ablest editors in the country said to me that he was in favor of anatomical articulation, provided the teeth were copper riveted to the jaws.—*Pacific Dental Gazette.*

